# Cord Blood Adiponectin and Visfatin Concentrations in relation to Oxidative Stress Markers in Neonates Exposed and Nonexposed* In Utero* to Tobacco Smoke

**DOI:** 10.1155/2016/4569108

**Published:** 2016-07-20

**Authors:** Magdalena Chełchowska, Jadwiga Ambroszkiewicz, Joanna Gajewska, Grażyna Rowicka, Tomasz M. Maciejewski, Joanna Mazur

**Affiliations:** ^1^Screening Department, Institute of Mother and Child, 01-211 Warsaw, Poland; ^2^Department of Nutrition, Institute of Mother and Child, 01-211 Warsaw, Poland; ^3^Department of Obstetrics and Gynaecology, Institute of Mother and Child, 01-211 Warsaw, Poland; ^4^Department of Child and Adolescent Health, Institute of Mother and Child, 01-211 Warsaw, Poland

## Abstract

*Aims.* Maternal smoking is considered as a source of oxidative stress, which has been implicated to disrupted adipokines expression in adipose tissue. We examined the relationship between selected adipokines and markers of oxidative stress/antioxidant defence in the umbilical cord of neonates exposed and nonexposed* in utero* to tobacco smoke.* Methods. *Subjects including 85 healthy neonates (born to 41 smokers and 44 nonsmokers) were tested for adiponectin, visfatin, oxidized low density lipoprotein (ox-LDL), total oxidant capacity (TOC), and total antioxidant capacity (TAC).* Results.* Cord serum visfatin, ox-LDL, and TOC were significantly higher (*p* < 0.001) but adiponectin and TAC were lower (*p* < 0.001 and *p* < 0.05, resp.) in smoking group than in tobacco abstinents. In whole group of children (adjusted for smoking status, gender, and birth weight) adiponectin showed negative and visfatin positive correlations with ox-LDL. In the model estimated separately for smokers ox-LDL explained 36% of adiponectin and 35.5% of visfatin variance, while in the model of nonsmokers it explained 36.8% and 69.4%, respectively.* Conclusion.* Maternal smoking enhances oxidative status and depletes antioxidant potential in newborns. Lower level of adiponectin and higher visfatin concentration seem to be related with a less beneficial oxidative stress profile and higher level of lipid peroxidation in neonates exposed and nonexposed* in utero* to tobacco smoke.

## 1. Introduction

Oxidative stress (OS) has been associated with numerous adverse pregnancy outcomes and fetal disturbances [1, 2]. Due to overproduction of reactive oxygen (ROS) and reactive nitrogen species (RNS) as well as inadequate induction of antioxidant protection newborns are particularly susceptible to oxidative injury [3, 4]. The oxidized LDL, which is considered as a strong indicator of oxidative stress, can stimulate placental endothelial dysfunction and lead to intrauterine growth retardation (IUGR) and low birth weight [5]. Furthermore, it has been shown that oxidative stress in adipose tissue may also impair neonatal condition in consequence of disrupted adipokines expression [6]. Adipokines are bioactive molecules expressed and secreted mainly from adipose tissue playing critical roles in energy homeostasis and are regarded to be key regulators of insulin sensitivity [7, 8]. Moreover, adipokines are constitutively expressed by the fetoplacental unit and are present in cord blood suggesting an involvement of these molecules in fetal development [9]. Adiponectin is a 244-amino-acid protein with a molecular weight of 28 kDa which modulates many metabolic processes, especially the metabolism of carbohydrates and fatty acids, indirectly affecting insulin resistance [10, 11]. Described potentially anti-inflammatory and antioxidative properties of adiponectin make it extremely interesting as an active factor in maintaining the balance between ROS generation and antioxidant defence production [12].* In vitro* experiment showed that adiponectin protects against endothelial dysfunction and cellular disruption induced by oxidized low density lipoprotein (ox-LDL) [13]. In an* in vivo* study significantly lower levels of lipid peroxidation products in adiponectin-overexpressing transgenic mice compared with wild-type mice were documented [14]. Also in a few human studies this association was studied, but the results are ambiguous [6, 15–17]. Visfatin originally identified as pre-B-cell colony-enhancing factor (PBEF) is a 491-amino-acid protein with molecular weight 52 kDa which is thought to have insulin-mimetic metabolic effects [18]. Additionally visfatin activates cytokines release and phospholipid metabolism; therefore, increasingly, it is considered as a proinflammatory adipokine [19, 20]. Contrary to adiponectin, high level of visfatin and increased level of lipid peroxidation products were shown in obese subjects and in patients with unstable carotid and coronary atherosclerosis [21].

Maternal smoking has been considered as an additional source of oxidant stress in pregnant women and in newborns exposed* in utero*, leading to perinatal and postnatal health consequences [22]. Cigarette smoke rich in free radicals and oxidizing species depletes plasma antioxidant defence and causes maternal chronic inflammation, decreased trophoblast invasion, and impaired placental metabolism and function [23]. Nicotine through higher secretion of catecholamines increased lipolysis and levels of plasma-free fatty acids resulting in higher concentrations of oxidatively modified lipid products in mothers as well as fetoplacental units [23, 24]. Oxidative stress and inflammatory process appear to be the main factors of endothelial dysfunction in smoking pregnant women which may result in the progression of insulin resistance [25]. Due to disorders of insulin sensitivity, changes in concentrations of adiponectin and visfatin in neonates of smoking mothers might be a physiological response to vascular endothelial damage in placental vasculature [6, 26]. Although smoking has been associated with increased oxidative stress in a number of studies, the finding that oxidative markers are independently associated with adipokines status in newborn of smoking pregnant women is relatively new [6, 23, 27–29]. Thus, in the present study, we sought to assess the association between selected adipokines (adiponectin and visfatin) and oxidative stress markers (ox-LDL, TOC, total oxidant status, and OSI, oxidative stress index) in cord blood of neonates exposed and nonexposed* in utero* to tobacco smoke. Relationships between total antioxidant status (TAC) and adiponectin as well as visfatin were also studied.

## 2. Material and Methods

The study was performed in accordance with Helsinki Declaration for Human Research and the study protocol was approved by the Ethics Committee of the Institute of Mother and Child in Warsaw, Poland. All mothers of participating infants were informed of the study objectives and written consent was obtained for analysis of cord blood samples and linking results to the data collected from questionnaires.

### 2.1. Subjects

The study was conducted in the Institute of Mother and Child in Warsaw between January 2012 and March 2014. Cord blood samples were obtained from 86 healthy women at delivery following a pregnancy of 37–42 weeks. The study included a consecutive series of 41 active smokers who smoked minimum 5 cigarettes per day throughout their pregnancy and smoked minimum 2 years before conception and a series of 45 nonsmokers of similar age and age of gestation, who had never smoked and were not exposed to environmental tobacco smoke during their pregnancy (smoking spouse or coworkers). History of smoking was obtained by direct questioning of the pregnant women and the classification was confirmed by measurement of serum cotinine concentration in mothers and their children. A cut-off value of ≥13.7 *μ*g/L was used to separate smokers from nonsmokers according to Jarvis et al. [30] who selected the optimal value which, in relation to self-reported smoking, misclassified the fewest subjects. This observation was confirmed in two studies in big population of pregnant women (3550 and 1134 participants) [31, 32]. In our study the high correlation (*r* = 0.9, *p* = 0.000) between maternal and fetal cotinine level was observed; therefore we decided to use the same cut-off value.

Gestational age at birth was estimated by the last menstrual period and confirmed by ultrasound measurements. Inclusion criteria were uncomplicated singleton pregnancies, birth weight appropriate for the gestational age, Apgar score of fifth minute more than 9 points, gestational age between 37 and 42 weeks, and spontaneous labor. The exclusion criteria for the study were maternal diseases (preeclampsia, hypertension, diabetes mellitus, active hepatitis, renal and cardiovascular diseases, and inflammatory conditions), multiple pregnancy, birth defects detected during pregnancy, assisted reproduction, delivery complications, and prolonged labor. All of subjects remained on a mixed diet and lived in an urban area. None of the mothers reported drinking alcohol and using drugs or illicit substances.

Prepregnancy body mass index (BMI) was calculated using height and prepregnancy weight (BMI = kg/m^2^). Newborn infants were evaluated in the first 24 hours of life. Neonatal length and weight were determined using a measuring board to the nearest 0.1 cm and a calibrated scale to the nearest 10 g.

### 2.2. Blood Sampling and Biochemical Analysis

Mixed venous and arterial umbilical cord blood samples (5 mL) were collected at the time of delivery from the umbilical vein before placental separation. In order to obtain serum, the blood was centrifuged at 2500 ×g, at 4°C for 10 minutes, and was stored in small portions for subsequent biochemical analysis.

Total oxidant capacity and total antioxidant capacity values were measured by colourimetric assay (Labor Diagnostika Nord GmbH&Co.KG, Nordhorn, Germany). The method is based on the enzymatic reaction of peroxides and peroxidases. Oxygen produced by this reaction oxidizes the chromogenic substrate tetramethylbenzidine (TMB), which changes its colour from colourless to blue. By addition of sulfuric acid the reaction cascade is stopped and the colour of mixture changes to yellow and can be detected at 450 nm. Serum peroxide levels were calculated as the difference of the absorbance readings relating to the hydrogen peroxide standard curve. Antioxidants inhibit this reaction and can be detected analogously on the basis of the indirect proportionality of this inhibition reaction. The limit of detection was 0.06 mmol/L for TOC and 0.08 mmol/L for TAC, respectively. The intra- and interassay coefficients of variation were less than 4.9% and 7.33% for TOC and 2.5% and 3.33% for TAC, respectively. Oxidative stress index (OSI) was defined as the percentage ratio of TOC levels to TAC levels.

ox-LDL levels were determined by enzyme-linked immunosorbent assay (ELISA) (Immundiagnostik AG, Bensheim, Germany). The intra- and interassay coefficients of variability were found less than 5.7% and 9.0%. The limit of detection was 4.13 ng/mL.

Total adiponectin and visfatin concentrations were determined by immunoassay (ELISA) (ALPCO Diagnostics, Salem, USA; Ray Biotech Inc., Norcross, USA, resp.). The intra- and interassay coefficients of variability were found less than 5.0% and 5.3% for adiponectin and 10% and 15.0% for visfatin. The limit of detection was 0.019 ng/mL for adiponectin and 0.778 ng/mL for visfatin, respectively.

Cotinine levels were evaluated by immunoenzymatic method using a commercially available kit (Calbiotech Inc., Spring Valley, CA, USA). The detection limit was 1.0 ng/mL.

### 2.3. Statistical Analysis

All of the statistical analyses were performed using SPSS statistical software version 17.1 (SPSS Inc., Chicago, IL, USA). The normality of data was tested using Kolmogorov-Smirnov test. The results were presented as means with standard deviation (SD) for normally distributed data or median with interquartile range (25th–75th percentiles) for nonnormally distributed variables (gestational age, Apgar score, number of cigarettes/day, and time of smoking before conception). In the smoking and nonsmoking groups, the baseline characteristics were compared using the Student *t*-test or Mann-Whitney *U* test depending on the assumptions. The Chi-squared test was used for comparing nominal variables. Correlations between the plasma adipokines (adiponectin and visfatin) and other normally distributed variables were assessed by Pearson's coefficient of correlation. Stepwise linear regression analysis was performed to study the relationship between adipokines level and oxidative stress markers. Independent variables were presented in the order of importance. Results were presented as the value of *B* unstandardized regression coefficient with 95% confidence interval and change in *R*-squared coefficient after each variable was entered. Models were estimated separately for smokers and nonsmokers as well as for total group. Three oxidative stress markers (ox-LDL, TOC, and TAC) were main independent variables. Regression models were adjusted for child gender and birth weight (all models: total, smokers, and nonsmokers group), for smoking status (total group), and for number of cigarettes (smokers). A *p* value <0.05 was considered statistically significant.

## 3. Results

The clinical characteristics of the study sample and biochemical findings of the subjects are given in Table 1. Nonsignificant differences were noted between the two groups with respect to clinical characteristics except for cigarette smoking habits. Birth weights and body length of the smokers' newborns were found to be lower than those of nonsmokers; however, in the case of length, there were no statistical differences. According to biochemical markers, the newborns of smoking mothers had significantly higher concentrations of serum visfatin, ox-LDL, TOC, and OSI (*p* < 0.001) but lower adiponectin (*p* < 0.05) and TAC (*p* < 0.001) levels compared with newborns of nonsmoking women.

In whole group we observed significant inverse correlation between studied adipokines (*p* < 0.001). Cord serum adiponectin was negatively associated with ox-LDL, TOC, and OSI levels and smoking status and positively correlated with TAC concentration and birth weight and length. On the contrary, cord serum visfatin was related positively with markers of oxidative stress (ox-LDL, TOC, and OSI) and negatively with TAC, smoking status, and anthropometric parameters (Table 2).

**Figure 1:**
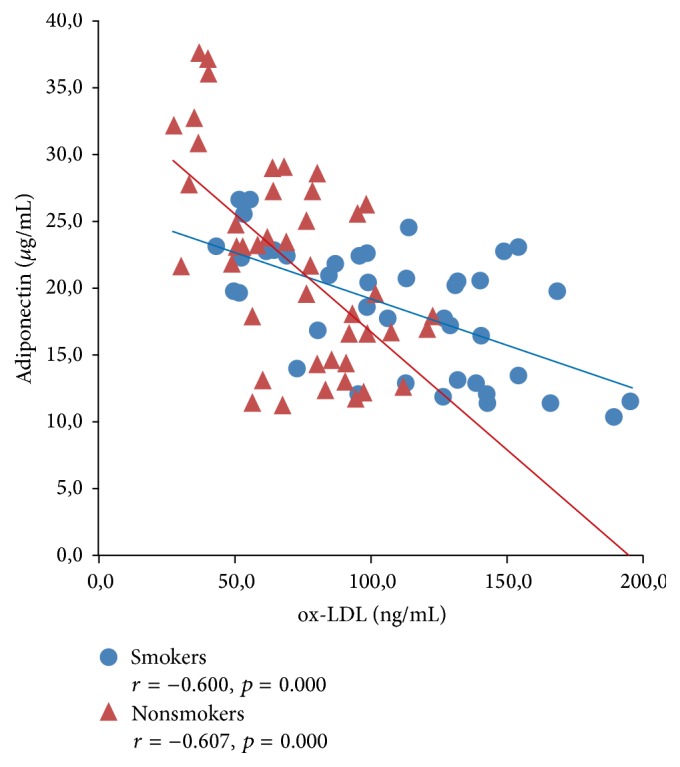
Correlations between cord serum adiponectin and ox-LDL levels in newborns of smokers and nonsmokers.

**Figure 2 fig2:**
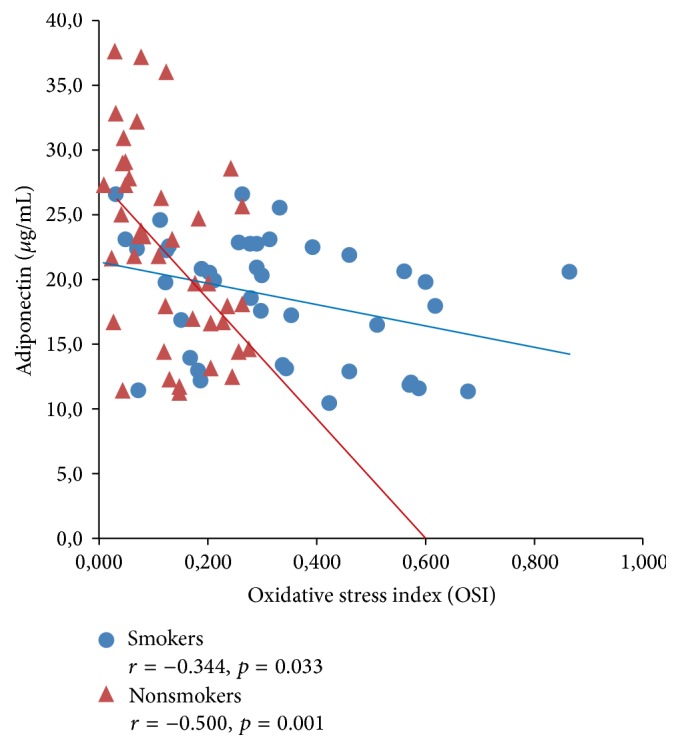
Correlations between cord serum adiponectin and OSI levels in newborns of smokers and nonsmokers.

**Figure 3 fig3:**
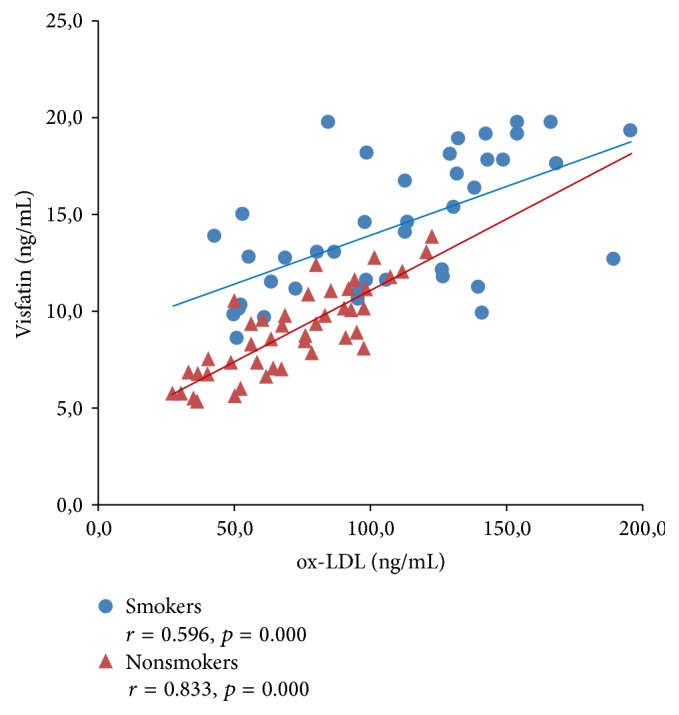
Correlations between cord serum visfatin and ox-LDL levels in newborns of smokers and nonsmokers.

**Figure 4:**
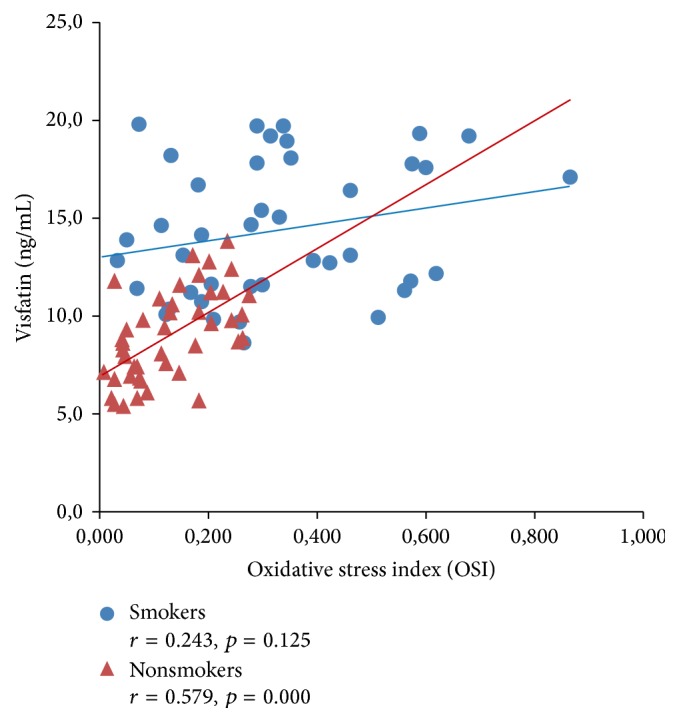
Correlations between cord serum visfatin and OSI levels in newborns of smokers and nonsmokers.

Figures 1 [Fig fig2] [Fig fig3]–4 expressed correlation between studied adipokine and oxidative stress markers separately for smokers and tobacco abstinent group. There was significant inverse relationship between adiponectin and ox-LDL as well as OSI level in both studied groups (Figures 1 and 2). Cord serum visfatin correlated positively with ox-LDL concentration in newborn of smoking and nonsmoking mothers, while association with OSI level was found only in tobacco abstinent group (Figures 3 and 4).

In multiple regression analysis performed in whole group (adjusted for smoking status, child gender, and birth weight) with ox-LDL, TOC, and TAC as independent variables, those associated with cord serum adiponectin were ox-LDL and birth weight; those associated with cord serum visfatin were ox-LDL and smoking status. In the model estimated separately for smokers (adjusted for child gender, birth weight, and number of cigarettes/day) the highest impact of the serum ox-LDL was indicated for adiponectin as well as visfatin levels. In these subjects ox-LDL explained 36% of adiponectin and 35.5% of visfatin variance. In the model of nonsmokers (adjusted for child gender and birth weight) ox-LDL, birth weight and TOC were of significant importance for adiponectin and ox-LDL for visfatin concentration. In those subjects ox-LDL explained 36.8% and 69.4% of adiponectin and visfatin variance, respectively (Table 3).

## 4. Discussion

The results of this study demonstrate a significant relationship between cord serum concentrations of adipokines and oxidative stress markers and confirmed the negative effect of tobacco smoking on these parameters. Pregnancy is a physiological state accompanied by high metabolic demand, changed glucose and lipid profile, and increased susceptibility to oxidative stress [23, 33]. In normal pregnancies, increased production of reactive oxygen species depletes antioxidant status in mothers, leading to oxidative stress in fetus, which may contribute to birth outcomes such as preterm birth, low birth weight, and developmental problems [1, 6, 34]. In women smoking during pregnancy, the risk of oxidative damage not only depends on the amount of physiologically enhanced ROS but can be affected also by intoxication with tobacco smoke radicals. Pregnant women who smoked had higher total oxidant status and concentration of lipid peroxidation products compared with tobacco abstinent [22, 23, 27, 28]. Exposure to cigarette smoke may also reduce the level of particular antioxidants and in consequence decreases total antioxidant capacity in the course of gestation [27, 35, 36].

There have not been many studies conducted concerning effect of maternal smoking on oxidant-antioxidant status in cord blood of newborns. No differences on MDA (malondialdehyde, breakdown product of lipid peroxidation) and antioxidant parameters as well as reduced level of total antioxidant capacity and increased levels of lipid peroxide products in cord blood of smokers were found [22, 28, 29, 35–37]. Because of the rapid metabolism and wide variation of MDA, we suggested that plasma ox-LDL level or TOC level could be a better marker of ROS activity in the fetus. In the presented study, conducted in group with confirmation of smoking status by cotinine levels, ox-LDL concentrations were higher in newborns of smoking mothers as compared with nonsmoking group. Increased susceptibility of LDL to oxidation in smoking group may be due to several reasons. One of them can be the raised amount of oxidizable substrate. Smokers have higher level of total cholesterol, LDL-cholesterol, and triglycerides [38, 39]. In addition, the pregnancy intensifies these changes [1]. As mentioned in Introduction, nicotine through higher secretion of catecholamines leads to intensification of lipolysis and increased free fatty acids, particularly PUFA (polyunsaturated fatty acids), a substrate for lipid oxidation [23, 24, 40]. Both phases of cigarette smoke (CS) contain high concentrations of oxidants and free radicals (estimated at 1 × 10^15^ radicals per puff) like superoxide anion (O_2_^∙−^), reactive hydroxyl radical (HO^*∙*^), H_2_O_2_, and nitric oxide (NO^*∙*^) which may directly influence the oxidation of serum LDL. A fast reaction between nitric oxide and superoxide anion produced the relatively long-lived potent prooxidant peroxynitrite anion (ONOO^−^), which is highly toxic and initiates lipid peroxidation [41]. Yamaguchi et al. [42] demonstrated* in vivo* and* in vitro* studies that oxidative modification of LDL induced by cigarette smoke can be mediated via peroxynitrite (measured by formation of 3-nitrotyrosine in plasma LDL). Some authors postulated mechanisms by which LDL becomes oxidized by CS with actions of several enzymes within the artery wall [42–45]. Increased expression of lipoxygenase which can be considered as a potential protein to oxidize LDL was observed in smokers [43]. Cigarette smoke and thiol-reactive substances found in CS induce O_2_^∙−^ and H_2_O_2 _production by activating NADPH oxidase in endothelial cells [44]. Smokers have also higher level of myeloperoxidase (MPO) which can be regarded as a “radical starter,” as it produces hypochlorous acid and in consequence might oxidize lipoproteins [45, 46]. Another important factor affecting the amount of ox-LDL may be insufficient concentrations of antioxidants. Cigarette smoking reduces the level of certain nonenzymatic antioxidants (glutathione, vitamins E and C, and *β*-carotene) as well as activity of free radical scavenging enzyme (glutathione peroxidase and superoxide dismutase) [22, 23, 27, 37]. Particularly vitamin E shows high effectiveness in preventing oxidation of LDL by cigarette smoke which was confirmed by other investigators [42, 45]. In our previous study we have shown the negative effect of maternal smoking on concentration of vitamin E, vitamin A, and *β*-carotene in umbilical cord blood [27].

In the present study we also evaluated the global oxidant/antioxidant status in newborns of smoking and nonsmoking mothers. For total oxidant capacity determination we used method described by Tatzber et al. [46] based on oxidation of TMB by horseradish peroxidase (HRP)/H_2_O_2_. This type of reaction is the basic mechanism for detecting both peroxide levels and peroxidase activity in blood serum. In the case of measurement of total peroxides HRP was added, while excess hydrogen peroxide was added for determination of enzyme activity [46]. Similar to Aycicek and Ipek [28] our findings showed significantly higher TOC and OSI levels in neonates exposed* in utero* to tobacco smoke compared with tobacco abstinent offspring. Because the above is accompanied with decreased levels of total antioxidant capacity, we speculate the severity of oxidative stress in this group.

It has been recently shown that level of oxidation products could be associated with some adipokine concentrations. These molecules participating in the regulation of glucose and lipids metabolism play a major role to promote optimal fetal development [7–9]. Increased serum adiponectin was assumed to be compensatory for early vascular endothelial damage and may have anti-inflammatory and antioxidative properties [6, 17]. On the contrary, visfatin can serve as a marker of insulin resistance with proinflammatory and prooxidant condition [8, 19, 47, 48]. This is particularly important due to the fact that smoking even in small amounts may cause endothelial dysfunction and transient uteroplacental vasoconstriction [6, 49]. Abnormal development of the placental vasculature leads to placental insufficiency which could compromise nutrient and gas transfer to the fetus and cause fetal growth restriction and low birth [49, 50]. There are only a few studies which explored the effect of maternal tobacco smoking on the concentrations of adiponectin and visfatin in cord blood [26, 51–53]. Similar to Pardo et al. [51] and our previous results [53], in the presented study adiponectin concentrations were significantly lower in umbilical cord blood of the smokers than in tobacco abstinent ones. On the contrary, Fang et al. [52] show no differences in adiponectin levels in cord blood between smokers and nonsmokers, but the study group of those authors had only 18 smokers based on self-reports and smoking means 5 cigarettes per day. In parallel to other investigators, we observed the positive correlation between adiponectin and birth weight [51]. López-Bermejo et al. [26] found increased cord serum visfatin in smaller babies of smoking mothers who smoked ten and more cigarettes per day. These authors showed that in the whole group cord serum visfatin was inversely associated with birth weight. Our results confirmed this observation.

To our knowledge, this is the first study to explore the effect of maternal smoking status on relations between oxidative stress markers and adiponectin as well as visfatin levels in newborns. Contrary to the study of Makedou et al. [6], we found negative correlations between adiponectin and ox-LDL in whole group of children (adjusted for smoking status, gender, and birth weight) as well as separately in smoking and nonsmoking group. Thus, Makadou et al. [6] conducted their research in a group of pregnant women, while fetal adipose tissue, rather than maternal production, may be a major source of adiponectin production in newborns [53]. Similar to our results, evidences from both humans and animals studies support the inverse correlations between oxidative stress and adiponectin level [15–17, 54]. Moreover, we observed negative effect of TOC and OSI on adiponectin level of our patients. These results, in conjunction with the positive relation with TAC, may confirm antioxidative role of this protein. In accordance with the findings of other authors, in our study visfatin correlated positively with ox-LDL, TOC, and OSI and negatively with TAC, which can suggest its prooxidative capacity [55, 56]. The lower level of visfatin coexisting with higher levels of TOC, OSI, and lipid hydroperoxide in women with premenstrual syndrome was observed [47]. The current study reported that in both the smoking and nonsmoking groups ox-LDL was a main predictor of adiponectin and visfatin concentrations in cord blood.

Recent study indicated that ox-LDL promotes the expression of visfatin through its activation of the endoplasmic reticulum (ER) stress in endothelial cells and macrophages [55]. Relationship between adiponectin and visfatin concentrations and ox-LDL production dependent on inflammation status was also observed [17, 21, 57]. As we described above, placenta is a site of active oxygen metabolism continuously generating oxidative stress. The placenta is also particularly susceptible to ER stress leading to poor placental development. Moreover, the activation of ER stress has been shown to induce inflammatory pathways [2, 24]. The adverse effect of nicotine in the placenta mediated through increased ER stress, inflammation, and oxidative stress was documented [24]. Therefore it is possible that tobacco smoking during pregnancy may affect adipokine levels by increased production of ox-LDL. Due to the transition from hypoxic intrauterine environment to extrauterine life, oxidative stress appears after birth in all newborns, regardless of the exposure to the tobacco smoke* in utero* [3]. Thus, as shown by us antioxidative property of adiponectin and prooxidant tendency of visfatin in cord blood cells of nonsmokers seem to be important for maintaining the oxidative-antioxidative balance in newborns.

The present study has some limitations. First, our sample is relatively of small size. However, both studied groups were similar for maternal BMI, age of gestation, type of delivery, and fetal gender, which are recognized as important factors for adipokine and oxidative stress marker levels [18, 20, 58–60]. Second, we cannot achieve the percentage of smokers representative of a normal pregnant population with the enrolment method we used. However, we were able to compare groups that did not differ in terms of their size and basic characteristics (listed above). The applied research scheme is not typical for a case-control study with retrospective assumptions [61]. This is a prospective comparison of cohorts exposed and not exposed to the effect of one factor, in this case tobacco smoking. Third, we did not measure any inflammatory markers in cord blood, but our study group consists of newborns of healthy pregnant women without inflammation process confirmed by negative c-reactive protein. Fourth, levels of cord serum lipids were not determined, but it is known for long time that maternal smoking during pregnancy markedly affects lipid metabolism in the fetus [39].

In conclusion, our data imply that maternal smoking enhances oxidative status and depletes antioxidant potential in newborns. Lower level of adiponectin and higher level of visfatin seem to be related with a less beneficial oxidative stress profile and higher level of lipid peroxidation in neonates exposed and nonexposed* in utero* to tobacco smoke.

## Figures and Tables

**Table 1:** Clinical characteristics and biochemical measurements of the study subjects (*N* = 85).

	Nonsmokers	Smokers	*p* value
	*N* = 44	*N* = 41	
*Newborn*			
^c^Male/female (%)	56.8/43.2	53.7/46.3	0.470
^b^Gestational age (week)	39 (39-40)	39 (38.5–40)	0.086
^b^Apgar score (5th min)	10 (10-10)	10 (10-10)	0.146
^a^Birth weight (g)	3511.4 ± 426.4	3123.7 ± 431.3	0.000
^a^Birth length (cm)	55.6 ± 2.7	54.4 ± 2.8	0.058
^a^Adiponectin (*µ*g/mL)	21.8 ± 7.4	18.6 ± 4.8	0.019
^a^Visfatin (ng/mL)	9.0 ± 2.3	14.4 ± 3.5	0.000
^a^ox-LDL (ng/mL)	71.7 ± 25.4	108.9 ± 41.1	0.000
^a^TOC (mmol/L)	0.195 ± 0.102	0.352 ± 0.164	0.000
^a^TAC (mmol/L)	1.694 ± 0.382	1.189 ± 0.277	0.000
^a^OSI	0.128 ± 0.080	0.323 ± 0.195	0.000
^a^Cotinine (*µ*g/L)	0	81.5 ± 29.3	—
*Mother*			
^a^Age (years)	28.9 ± 4.7	28.2 ± 4.4	0.465
^a^Maternal weight (kg)	64.8 ± 5.5	63.8 ± 5.2	0.398
^a^Maternal height (cm)	164.6 ± 4.5	165.1 ± 4.9	0.651
^a^Pregravid BMI (kg/m^2^)	23.9 ± 1.4	23.4 ± 1.43	0.095
^b^Number of cigarettes/day	0	10 [5–10]	—
^a^Cotinine (*µ*g/L)	0	89.2 ± 30.4	—

^a^Values are means ± standard deviation (SD), ^b^values are median and interquartile range (25th–75th percentiles), and ^c^values are percentage. ox-LDL: oxidized low density lipoprotein; TOC: total oxidant capacity; TAC: total antioxidant capacity; OSI: oxidative stress index; BMI: body mass index.

**Table 2:** The correlations between adipokines (adiponectin, visfatin) and clinical/biochemical parameters in studied subjects (*N* = 85).

	Adiponectin		Visfatin	
	*r*	*p* value	*r*	*p* value
Gender	0.096	0.384	0.028	0.799
Gestational age	0.203	0.062	−0.147	0.180
Birth weight	0.444	0.000	−0.377	0.000
Birth length	0.322	0.003	−0.223	0.040
Apgar	−0.039	0.720	−0.061	0.577
Adiponectin	—	—	−0.445	0.000
Visfatin	−0.445	0.000	—	—
ox-LDL	−0.582	0.000	0.758	0.000
TOC	−0.439	0.000	0.558	0.000
TAC	0.457	0.000	−0.586	0.000
OSI	−0.403	0.000	0.570	0.000
Newborn cotinine	−0.287	0.008	0.658	0.000
Maternal age	0.154	0.160	−0.050	0.651
Maternal weight	0.036	0.745	0.033	0.765
Maternal height	0.000	0.999	0.109	0.323
Maternal BMI	0.056	0.608	−0.058	0.600
Smoking status (no = 0; yes = 1)	−0.251	0.021	0.683	0.000

ox-LDL: oxidized low density lipoprotein; TOC: total oxidant capacity; TAC: total antioxidant capacity; OSI: oxidative stress index; BMI: body mass index.

**Table 3:** Multivariable regressions of adiponectin and visfatin with markers of oxidative status (ox-LDL, TOC, and TAC) as independent variables.

Dependent and independent variables	*B*	95% CI	*p* value	Δ*R* ^2^
Dependent variable: adiponectin, *N* = 85^*∗*^				
ox-LDL	−0.081	−0.112/−0.049	0.000	0.339
Birth weight	0.003	0.001/0.006	0.009	0.053
Dependent variable: visfatin, *N* = 85^*∗*^				
ox-LDL	0.057	0.043/0.0711	0.000	0.574
Smoking status	−3.243	−4.314/−2.171	0.000	0.130
Dependent variable: adiponectin, *N* = 41 (smokers)^*∗∗*^				
ox-LDL	−0.069	−0.099/−0.039	0.000	0.360
Dependent variable: visfatin, *N* = 41 (smokers)^*∗∗*^				
ox-LDL	0.050	0.028/0.072	0.000	0.355
Dependent variable: adiponectin, *N* = 44 (nonsmokers)^*∗∗∗*^				
ox-LDL	−0.110	−0.190/−0.031	0.008	0.368
Birth weight	0.006	0.002/0.010	0.008	0.083
TOC	−20.528	−39.604/−1.451	0.036	0.058
Dependent variable: visfatin, *N* = 44 (nonsmokers)^*∗∗∗*^				
ox-LDL	0.074	0.059/0.089	0.000	0.694

^*∗*^Adjusted for infant gender and birth weight and mother smoking status.

^*∗∗*^Adjusted for infant gender and birth weight and number of cigarettes.

^*∗∗∗*^Adjusted for infant gender and birth weight.
